# *N*′-(5-Bromo-2-methoxy­benzyl­idene)-2-hydroxy­benzohydrazide

**DOI:** 10.1107/S1600536808030900

**Published:** 2008-09-30

**Authors:** Jiu-Fu Lu

**Affiliations:** a School of Chemistry and Environmental Science, Shaanxi University of Technology, Hanzhong 723000, People’s Republic of China

## Abstract

The title Schiff base compound, C_15_H_13_BrN_2_O_3_, is derived from the condensation of 5-bromo-2-methoxy­benzaldehyde with 2-hydroxy­benzohydrazide in an ethanol solution. The dihedral angle between the two aromatic rings is 6.9 (9)°. The meth­oxy group is coplanar with the attached ring [C—O—C—C = 3.1 (12)°]. An intra­molecular N—H⋯O hydrogen bond is observed. In the crystal structure, the mol­ecules are linked into chains along the [001] direction by inter­molecular O—H⋯N, O—H⋯O and C—H⋯O hydrogen bonds.

## Related literature

For related structures, see: Lu *et al.* (2008*a* ▶,*b* ▶,*c* ▶); Nie (2008 ▶); He (2008 ▶); Shi *et al.* (2007 ▶). For bond-length data, see: Allen *et al.* (1987 ▶).
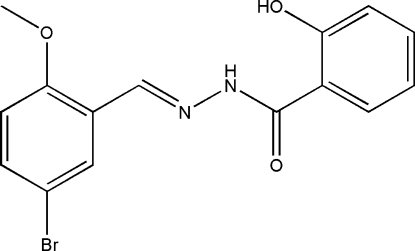


## Experimental

### 

#### Crystal data

C_15_H_13_BrN_2_O_3_
*M* *_r_* = 349.18
Tetragonal, 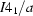

*a* = 15.530 (3) Å
*c* = 25.308 (2) Å
*V* = 6103.8 (17) Å^3^
*Z* = 16
Mo *K*α radiation
μ = 2.70 mm^−1^
*T* = 298 (2) K
0.12 × 0.10 × 0.10 mm

#### Data collection

Bruker APEXII CCD area-detector diffractometer
Absorption correction: multi-scan (*SADABS*; Sheldrick, 2004 ▶) *T*_min_ = 0.737, *T*_max_ = 0.774
25116 measured reflections
3322 independent reflections
1243 reflections with *I* > 2σ(*I*)
*R*_int_ = 0.128

#### Refinement

*R*[*F* ^2^ > 2σ(*F* ^2^)] = 0.068
*wR*(*F* ^2^) = 0.226
*S* = 0.99
3322 reflections
195 parameters
1 restraint
H atoms treated by a mixture of independent and constrained refinement
Δρ_max_ = 0.97 e Å^−3^
Δρ_min_ = −0.86 e Å^−3^

### 

Data collection: *APEX2* (Bruker, 2004 ▶); cell refinement: *SAINT* (Bruker, 2004 ▶); data reduction: *SAINT*; program(s) used to solve structure: *SHELXS97* (Sheldrick, 2008 ▶); program(s) used to refine structure: *SHELXL97* (Sheldrick, 2008 ▶); molecular graphics: *SHELXTL* (Sheldrick, 2008 ▶); software used to prepare material for publication: *SHELXTL*.

## Supplementary Material

Crystal structure: contains datablocks global, I. DOI: 10.1107/S1600536808030900/ci2679sup1.cif

Structure factors: contains datablocks I. DOI: 10.1107/S1600536808030900/ci2679Isup2.hkl

Additional supplementary materials:  crystallographic information; 3D view; checkCIF report

## Figures and Tables

**Table 1:** Hydrogen-bond geometry (Å, °)

*D*—H⋯*A*	*D*—H	H⋯*A*	*D*⋯*A*	*D*—H⋯*A*
N2—H2⋯O3	0.90 (3)	1.95 (5)	2.606 (6)	129 (5)
O3—H3⋯N1^i^	0.82	2.56	3.159 (6)	130
O3—H3⋯O2^i^	0.82	1.81	2.590 (5)	157
C6—H6⋯O3^ii^	0.93	2.55	3.471 (8)	174

Symmetry codes: (i) 
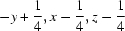
; (ii) 
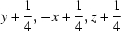
.

**Fig. 1.**
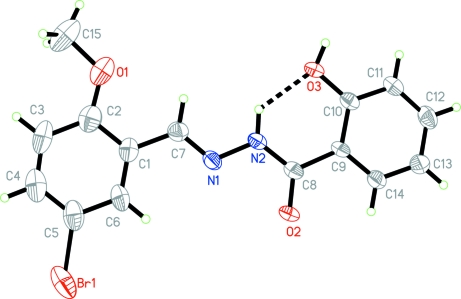
The molecular structure of the title compound, showing 30% probability displacement ellipsoids and the atom-numbering scheme. The intramolecular hydrogen bond is shown by a dashed line.

**Fig. 2.**
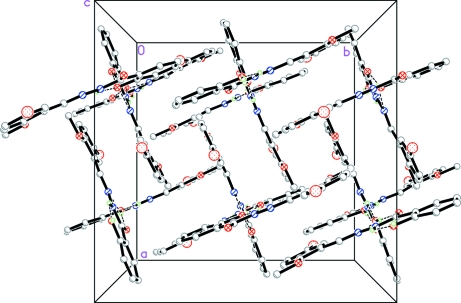
The crystal packing of the title compound, viewed along the c axis. H atoms not involved in hydrogen bonding (dashed lines) have been omitted for clarity.

## supplementary crystallographic information

### Comment

As part of our investigation of the crystal structures of Schiff bases derived from the condensation of aldehydes with benzohydrazides (Lu *et al.*, 2008*a*,b,c), we report here the crystal structure of the title new Schiff base compound.

In the title molecule (Fig. 1), the bond lengths have normal values (Allen *et al.*,  1987), and are comparable to those observed in similar compounds (Nie, 2008; He, 2008; Shi *et al.*,  2007). The methoxy group is coplanar with the attached ring [C15—O1—C2—C3 = 3.1 (12)°]. The dihedral angle between the two aromatic rings is 6.9 (9)°, indicating that the molecule is approximately planar. An intramolecular N—H···O hydrogen bond is observed in the molecule (Table 1).

In the crystal structure, the molecules are linked into chains (Fig. 2) along the [001] by intermolecular O—H···N, O—H···O and C—H···O hydrogen bonds (Table 1).

### Experimental

The title compound was prepared by the Schiff base condensation of 5-bromo-2-methoxybenzaldehyde (0.1 mol) and 2-hydroxybenzohydrazide (0.1 mol) in 95% ethanol (50 ml). The excess ethanol was removed by distillation. The colourless solid obtained was filtered and washed with ethanol. Single crystals suitable for X-ray diffraction were obatined by slow evaporation of a 95% ethanol solution at room temperature.

### Refinement

The imino H atom was located in a difference map and refined with a N-H distance restraint of 0.90 (1) Å and a fixed U_iso_ of 0.08 Å^2^. The other H atoms were positioned geometrically (C-H = 0.93-0.96 Å, O-H = 0.82 Å) and refined using a riding model, with U_iso_(H) = 1.2U_eq_(C) and 1.5U_eq_(C_methyl_ and O). A rotating group model was used for methyl and hydroxyl groups.The ratio of observed to unique reflections is low (37%), which is due to the poor diffraction quality of the crystal.

### Crystal data

**Table:**

C_15_H_13_BrN_2_O_3_	*D*_x_ = 1.520 Mg m^−^^3^
*M**_r_* = 349.18	Mo *K*α radiation, λ = 0.71073 Å
Tetragonal, *I*4_1_/*a*	Cell parameters from 1836 reflections
Hall symbol: -I 4ad	θ = 2.4–24.3°
*a* = 15.530 (3) Å	µ = 2.70 mm^−^^1^
*c* = 25.308 (2) Å	*T* = 298 K
*V* = 6103.8 (17) Å^3^	Block, colourless
*Z* = 16	0.12 × 0.10 × 0.10 mm
*F*(000) = 2816	

### Data collection

**Table:**

Bruker APEXII CCD area-detector diffractometer	3322 independent reflections
Radiation source: fine-focus sealed tube	1243 reflections with *I* > 2σ(*I*)
graphite	*R*_int_ = 0.128
ω scans	θ_max_ = 27.0°, θ_min_ = 1.5°
Absorption correction: multi-scan (SADABS; Sheldrick, 2004)	*h* = −19→19
*T*_min_ = 0.737, *T*_max_ = 0.774	*k* = −19→19
25116 measured reflections	*l* = −32→32

### Refinement

**Table:**

Refinement on *F*^2^	Primary atom site location: structure-invariant direct methods
Least-squares matrix: full	Secondary atom site location: difference Fourier map
*R*[*F*^2^ > 2σ(*F*^2^)] = 0.068	Hydrogen site location: inferred from neighbouring sites
*wR*(*F*^2^) = 0.226	H atoms treated by a mixture of independent and constrained refinement
*S* = 0.99	*w* = 1/[σ^2^(*F*_o_^2^) + (0.0861*P*)^2^ + 12.6195*P*] where *P* = (*F*_o_^2^ + 2*F*_c_^2^)/3
3322 reflections	(Δ/σ)_max_ = 0.001
195 parameters	Δρ_max_ = 0.97 e Å^−^^3^
1 restraint	Δρ_min_ = −0.86 e Å^−^^3^

### Special details

**Table:**

Geometry. All esds (except the esd in the dihedral angle between two l.s. planes) are estimated using the full covariance matrix. The cell esds are taken into account individually in the estimation of esds in distances, angles and torsion angles; correlations between esds in cell parameters are only used when they are defined by crystal symmetry. An approximate (isotropic) treatment of cell esds is used for estimating esds involving l.s. planes.
Refinement. Refinement of F^2^ against ALL reflections. The weighted R-factor wR and goodness of fit S are based on F^2^, conventional R-factors R are based on F, with F set to zero for negative F^2^. The threshold expression of F^2^ > 2sigma(F^2^) is used only for calculating R-factors(gt) etc. and is not relevant to the choice of reflections for refinement. R-factors based on F^2^ are statistically about twice as large as those based on F, and R- factors based on ALL data will be even larger.

### Fractional atomic coordinates and isotropic or equivalent isotropic displacement parameters (Å^2^)

**Table:**

	*x*	*y*	*z*	*U*_iso_*/*U*_eq_	
Br1	0.13353 (7)	0.23636 (7)	0.44885 (4)	0.1335 (6)	
O1	0.0993 (4)	0.3108 (3)	0.2169 (3)	0.1107 (18)	
O2	0.2734 (3)	−0.0626 (3)	0.28327 (15)	0.0760 (13)	
O3	0.2429 (3)	−0.0129 (3)	0.12353 (14)	0.0729 (13)	
H3	0.2527	−0.0072	0.0919	0.109*	
N1	0.2045 (3)	0.0897 (4)	0.26121 (18)	0.0624 (14)	
N2	0.2295 (4)	0.0325 (3)	0.22229 (18)	0.0642 (14)	
C1	0.1451 (4)	0.2232 (4)	0.2860 (3)	0.0715 (19)	
C2	0.1073 (5)	0.3000 (6)	0.2705 (4)	0.090 (2)	
C3	0.0784 (6)	0.3586 (5)	0.3092 (5)	0.110 (3)	
H3A	0.0524	0.4101	0.2993	0.132*	
C4	0.0888 (6)	0.3391 (7)	0.3611 (5)	0.114 (3)	
H4	0.0705	0.3780	0.3867	0.137*	
C5	0.1251 (5)	0.2647 (6)	0.3760 (4)	0.096 (3)	
C6	0.1538 (5)	0.2065 (4)	0.3385 (3)	0.080 (2)	
H6	0.1794	0.1554	0.3493	0.095*	
C7	0.1749 (4)	0.1611 (5)	0.2463 (3)	0.0705 (19)	
H7	0.1720	0.1744	0.2105	0.085*	
C8	0.2622 (4)	−0.0430 (4)	0.2367 (2)	0.0581 (16)	
C9	0.2842 (4)	−0.1056 (4)	0.19439 (19)	0.0512 (15)	
C10	0.2728 (4)	−0.0908 (4)	0.1401 (2)	0.0572 (16)	
C11	0.2912 (4)	−0.1555 (5)	0.1043 (2)	0.0662 (18)	
H11	0.2822	−0.1467	0.0684	0.079*	
C12	0.3224 (5)	−0.2320 (5)	0.1218 (3)	0.079 (2)	
H12	0.3352	−0.2752	0.0976	0.095*	
C13	0.3354 (5)	−0.2465 (5)	0.1747 (3)	0.084 (2)	
H13	0.3582	−0.2987	0.1862	0.100*	
C14	0.3148 (4)	−0.1842 (4)	0.2101 (2)	0.0687 (18)	
H14	0.3217	−0.1952	0.2460	0.082*	
C15	0.0626 (6)	0.3886 (6)	0.1961 (5)	0.143 (4)	
H15A	0.0062	0.3968	0.2107	0.215*	
H15B	0.0585	0.3842	0.1583	0.215*	
H15C	0.0985	0.4366	0.2052	0.215*	
H2	0.221 (4)	0.050 (4)	0.1888 (9)	0.080*	

### Atomic displacement parameters (Å^2^)

**Table:**

	*U*^11^	*U*^22^	*U*^33^	*U*^12^	*U*^13^	*U*^23^
Br1	0.1547 (11)	0.1402 (10)	0.1057 (8)	−0.0250 (7)	0.0402 (6)	−0.0549 (6)
O1	0.108 (5)	0.077 (4)	0.147 (6)	0.014 (3)	−0.005 (4)	0.014 (4)
O2	0.113 (4)	0.083 (3)	0.032 (2)	0.018 (3)	0.002 (2)	0.002 (2)
O3	0.107 (4)	0.078 (3)	0.034 (2)	0.019 (3)	0.007 (2)	0.004 (2)
N1	0.081 (4)	0.059 (4)	0.048 (3)	−0.004 (3)	0.012 (3)	−0.006 (3)
N2	0.092 (4)	0.063 (4)	0.038 (3)	0.006 (3)	0.002 (3)	−0.004 (3)
C1	0.063 (5)	0.061 (5)	0.090 (6)	−0.003 (4)	0.013 (4)	−0.004 (4)
C2	0.078 (5)	0.073 (6)	0.120 (7)	−0.016 (5)	0.006 (5)	0.001 (6)
C3	0.092 (7)	0.057 (5)	0.181 (10)	0.005 (4)	0.019 (7)	−0.010 (7)
C4	0.107 (7)	0.085 (8)	0.150 (10)	−0.009 (6)	0.036 (7)	−0.034 (7)
C5	0.085 (6)	0.075 (6)	0.129 (7)	−0.016 (5)	0.026 (5)	−0.026 (5)
C6	0.082 (5)	0.066 (5)	0.091 (6)	−0.012 (4)	0.020 (4)	−0.025 (4)
C7	0.085 (5)	0.065 (5)	0.061 (4)	−0.004 (4)	0.007 (3)	0.005 (4)
C8	0.066 (4)	0.065 (5)	0.043 (4)	−0.007 (3)	0.007 (3)	−0.001 (3)
C9	0.061 (4)	0.058 (4)	0.034 (3)	−0.009 (3)	0.005 (3)	−0.001 (3)
C10	0.055 (4)	0.072 (5)	0.045 (3)	−0.005 (3)	0.008 (3)	−0.001 (3)
C11	0.074 (5)	0.075 (5)	0.049 (4)	−0.007 (4)	0.008 (3)	−0.008 (4)
C12	0.103 (6)	0.062 (5)	0.073 (5)	−0.007 (4)	0.017 (4)	−0.020 (4)
C13	0.116 (6)	0.067 (5)	0.067 (5)	0.018 (4)	0.015 (4)	0.011 (4)
C14	0.089 (5)	0.066 (5)	0.052 (4)	0.006 (4)	0.013 (3)	0.002 (3)
C15	0.109 (7)	0.100 (7)	0.221 (12)	0.011 (6)	−0.014 (7)	0.048 (7)

### Geometric parameters (Å, °)

**Table:**

Br1—C5	1.900 (10)	C5—C6	1.385 (10)
O1—C2	1.373 (10)	C6—H6	0.93
O1—C15	1.436 (9)	C7—H7	0.93
O2—C8	1.230 (7)	C8—C9	1.485 (8)
O3—C10	1.362 (7)	C9—C14	1.370 (8)
O3—H3	0.82	C9—C10	1.405 (7)
N1—C7	1.259 (7)	C10—C11	1.383 (8)
N1—N2	1.381 (7)	C11—C12	1.356 (9)
N2—C8	1.330 (7)	C11—H11	0.93
N2—H2	0.90 (3)	C12—C13	1.373 (10)
C1—C6	1.360 (10)	C12—H12	0.93
C1—C2	1.386 (10)	C13—C14	1.357 (9)
C1—C7	1.467 (9)	C13—H13	0.93
C2—C3	1.411 (12)	C14—H14	0.93
C3—C4	1.356 (13)	C15—H15A	0.96
C3—H3A	0.93	C15—H15B	0.96
C4—C5	1.340 (12)	C15—H15C	0.96
C4—H4	0.93		
			
C2—O1—C15	120.1 (8)	O2—C8—N2	122.4 (5)
C10—O3—H3	109.5	O2—C8—C9	119.7 (6)
C7—N1—N2	117.0 (5)	N2—C8—C9	117.9 (5)
C8—N2—N1	118.6 (5)	C14—C9—C10	118.3 (5)
C8—N2—H2	125 (4)	C14—C9—C8	117.0 (5)
N1—N2—H2	116 (4)	C10—C9—C8	124.7 (6)
C6—C1—C2	118.7 (7)	O3—C10—C11	121.0 (5)
C6—C1—C7	120.9 (7)	O3—C10—C9	119.3 (5)
C2—C1—C7	120.4 (8)	C11—C10—C9	119.7 (6)
O1—C2—C1	115.0 (8)	C12—C11—C10	119.7 (6)
O1—C2—C3	125.4 (9)	C12—C11—H11	120.1
C1—C2—C3	119.6 (9)	C10—C11—H11	120.1
C4—C3—C2	119.3 (9)	C11—C12—C13	121.1 (6)
C4—C3—H3A	120.3	C11—C12—H12	119.5
C2—C3—H3A	120.3	C13—C12—H12	119.5
C5—C4—C3	121.1 (9)	C14—C13—C12	119.4 (7)
C5—C4—H4	119.5	C14—C13—H13	120.3
C3—C4—H4	119.5	C12—C13—H13	120.3
C4—C5—C6	120.3 (9)	C13—C14—C9	121.8 (6)
C4—C5—Br1	120.2 (8)	C13—C14—H14	119.1
C6—C5—Br1	119.5 (8)	C9—C14—H14	119.1
C1—C6—C5	121.0 (8)	O1—C15—H15A	109.5
C1—C6—H6	119.5	O1—C15—H15B	109.5
C5—C6—H6	119.5	H15A—C15—H15B	109.5
N1—C7—C1	119.2 (6)	O1—C15—H15C	109.5
N1—C7—H7	120.4	H15A—C15—H15C	109.5
C1—C7—H7	120.4	H15B—C15—H15C	109.5

### Hydrogen-bond geometry (Å, °)

**Table:**

*D*—H···*A*	*D*—H	H···*A*	*D*···*A*	*D*—H···*A*
N2—H2···O3	0.90 (3)	1.95 (5)	2.606 (6)	129 (5)
O3—H3···N1^i^	0.82	2.56	3.159 (6)	130
O3—H3···O2^i^	0.82	1.81	2.590 (5)	157
C6—H6···O3^ii^	0.93	2.55	3.471 (8)	174

Symmetry codes: (i) −*y*+1/4, *x*−1/4, *z*−1/4; (ii) *y*+1/4, −*x*+1/4, *z*+1/4.
